# Sarcoidosis‐Like Granulomatous Reaction During Adjuvant Pembrolizumab for High‐Risk Resected Melanoma

**DOI:** 10.1155/crom/9633746

**Published:** 2026-06-10

**Authors:** Icíar De La Fuente DomÃnguez, Pedro Sánchez Mauriño, Beatriz Teso Martín, Enrique Aranda Aguilar

**Affiliations:** ^1^ University of Córdoba, Maimonides Biomedical Research Institute of Cordoba (IMIBIC), Reina Sofia University Hospital, Córdoba, Spain, juntadeandalucia.es; ^2^ Medical Oncology Department, Reina Sofia University Hospital, Córdoba, Spain, juntadeandalucia.es

**Keywords:** immune-related adverse events, immunotherapy, melanoma, PD-1 inhibitors, pembrolizumab, sarcoidosis-like reaction

## Abstract

**Introduction:**

Immunotherapy with immune checkpoint inhibitors has transformed the management of advanced melanoma and resected high‐risk disease. However, their use is associated with immune‐related adverse events, including sarcoidosis‐like granulomatous reactions—an uncommon toxicity that may be mimic disease progression or infection.

**Clinical Case:**

We report the case of a 70‐year‐old woman with Stage IIC cutaneous melanoma (pT4bN0M0), harboring an NRAS Q61L mutation, treated with adjuvant pembrolizumab. After three cycles, she developed subcutaneous nodules, lower‐limb edema, and ocular symptoms. Histological evaluation confirmed a sarcoidosis‐like granulomatous reaction. Pembrolizumab was discontinued and systemic corticosteroid therapy was initiated, resulting in partial clinical improvement.

**Discussion:**

Sarcoidosis‐like reactions (SLRs) are rare but clinically relevant adverse events associated with PD‐1 inhibitors. Their differential diagnosis is challenging and requires exclusion of tumor progression and infectious processes. These reactions should be considered in patients receiving immunotherapy who develop atypical systemic manifestations. This phenomenon may have prognostic implications and warrants multidisciplinary management approach.

**Conclusion:**

This case highlights the importance of recognizing immune‐mediated SLRs during immunotherapy, particularly in melanoma, and underscores the need for a high index of suspicion and further evidence to guide optimal management strategies.

## 1. Introduction

Cutaneous melanoma is a malignant tumor derived from melanocytes and accounts for approximately 1% of all skin cancers, while being responsible for the majority of skin cancer–related deaths due to its high propensity for lymphatic and hematogenous dissemination.

Its incidence has increased steadily over recent decades, representing a major global public health concern [1]. Prognosis is closely related to clinical stage at diagnosis, with Breslow thickness, ulceration, and nodal involvement being the main determinants of survival [2].

Immune checkpoint inhibitors, such as pembrolizumab, have been approved as adjuvant treatment for patients with resected high‐risk melanoma, leading to significant improvements in recurrence‐free survival [3]. Nevertheless, these agents are associated with immune‐related adverse events (irAEs), including granulomatous inflammatory conditions such as SLRs.

SLRs are inflammatory responses that share clinical and histological features with sarcoidosis, without necessarily fulfilling its classic diagnostic criteria [4]. They are characterized by the formation of noncaseating granulomas in multiple organs, mimicking systemic sarcoidosis. These reactions have been described as complications associated with PD‐1 and CTLA‐4 inhibitors, primarily in melanoma, nonsmall cell lung cancer, and renal cell carcinoma [5]. Early recognition is essential to avoid unnecessary interruption of oncologic treatment.

Recent advances in melanoma diagnosis and treatment have been comprehensively reviewed, highlighting the evolving role of immunotherapy and the complexity of managing associated toxicities, including irAEs [6].

## 2. Case Presentation

A 70‐year‐old woman with a medical history of hypertension, diabetes mellitus, and prior surgery for L5–S1 spinal stenosis was referred to dermatology in July 2024 due to the appearance of a pearly lesion on her left arm. An excisional biopsy revealed cutaneous melanoma staged as pT4bN0M0 (Stage IIC), PD − L1 expression < 1*%* by immunohistochemistry, BRAF V600E wild‐type, and NRAS Q61L mutation. Her ECOG performance status at diagnosis was 1.

After multidisciplinary tumor board discussion, wide local excision and sentinel lymph node biopsy were performed in October 2024, confirming tumor‐free surgical margins and no nodal involvement.

Adjuvant treatment with pembrolizumab 200 mg was initiated in December 2024. After the third cycle, the patient developed painful subcutaneous nodules on the arms and gluteal region, lower limb edema, and periocular irritation (Figures 1 and 2). Pembrolizumab was suspended, systemic corticosteroid therapy was initiated, and further diagnostic evaluation was undertaken.

**Figure 1:**
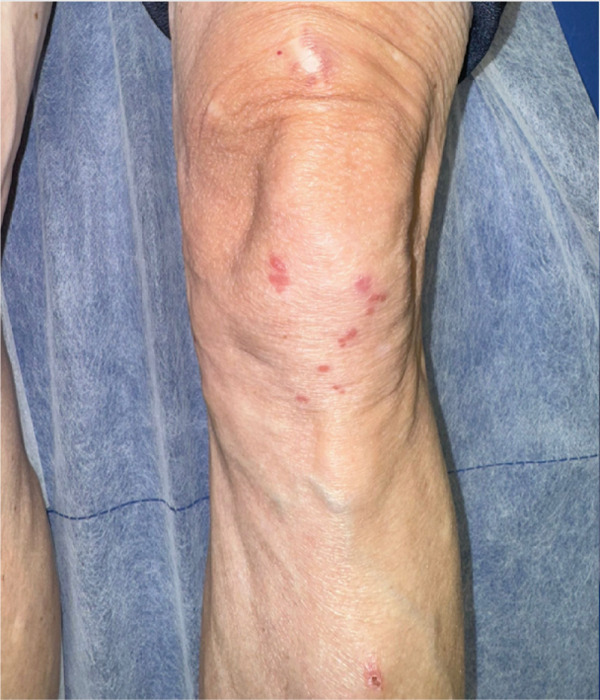
Subcutaneous nodules.

**Figure 2:**
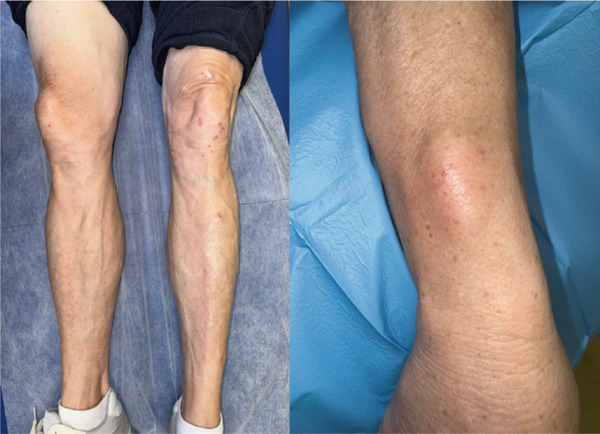
Subcutaneous nodules.

Ultrasound examination showed a poorly defined hypoechoic area within the superficial dermal and subcutaneous layers, without nodular features and with mild vascularity. These findings were nonspecific, suggesting focal panniculitis; however, the superficial and palpable nature of the lesions made ultrasound‐guided biopsy technically challenging.

A skin biopsy performed by dermatology revealed a dermal granulomatous reaction consistent with a sarcoidosis‐like process, most likely associated with pembrolizumab therapy. Computed tomography demonstrated hilar and mediastinal lymphadenopathy and perilymphatic pulmonary micronodules, findings suggestive of a treatment‐related sarcoid reaction (Figure 3). Serum angiotensin‐converting enzyme (ACE) levels were not assessed. No clinical or imaging findings suggestive of cardiac involvement were identified.

**Figure 3:**
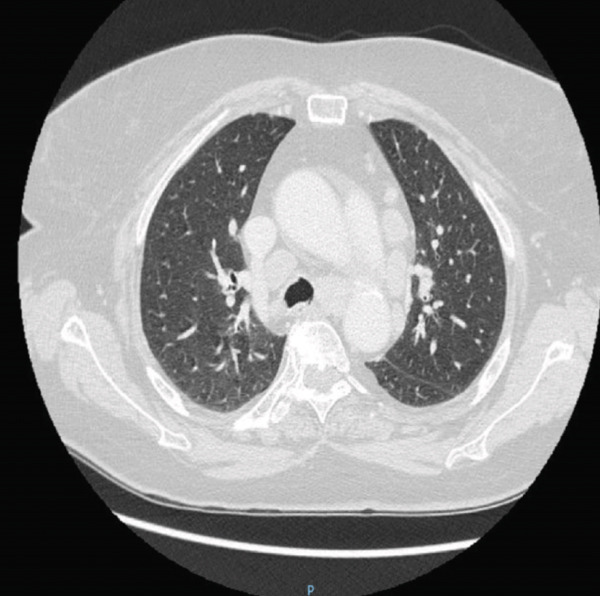
Computed tomography: hilar and mediastinal lymphadenopathy and perilymphatic pulmonary micronodules.

By March 2025, the patient continued to experience painful subcutaneous lesions and persistent ocular symptoms, including irritation, burning, and pruritus in both eyes. Resumption of adjuvant therapy was therefore ruled out, and she was referred to ophthalmology and internal medicine for multidisciplinary evaluation.

## 3. Discussion

Following diagnosis and surgical management of Stage IIC melanoma (pT4bN0M0), the patient initiated adjuvant pembrolizumab therapy. Immune checkpoint inhibitors have revolutionized the treatment of advanced melanoma, improving overall and progression‐free survival even in locally advanced disease. Pembrolizumab has demonstrated significant benefit in recurrence‐free survival in trials such as KEYNOTE‐054 in patients with resected Stage III melanoma, with benefits extrapolated to stage IIC disease in clinical practice [3].

After three treatment cycles, the patient developed subcutaneous nodules, ocular irritation, and lower limb edema and was subsequently diagnosed with a sarcoidosis‐like granulomatous reaction. Although uncommon, pembrolizumab‐induced SLRs have been reported in the literature, most frequently in patients treated for melanoma or lung cancer [7], and may occur at any time during treatment.

Recent studies indicate that these reactions typically manifest as noncaseating granulomas affecting the skin, lungs, eyes, and lymph nodes, and less commonly the liver or spleen. Diagnosis relies on histological confirmation and imaging studies. Typical CT findings include symmetric lymphadenopathy and peribronchovascular micronodules, which may closely resemble tumor progression, lymphoma, or infectious processes [4].

In addition, case series have described SLRs in melanoma patients receiving immunotherapy or targeted therapy, reinforcing the need for awareness of this entity in clinical practice [8].

The underlying mechanism is thought to involve dysregulated immune activation induced by inhibition of the PD‐1/PD‐L1 pathway, leading to an exaggerated T Helper 1 (Th1)–mediated immune response and subsequent granuloma formation. In addition, emerging evidence suggests a relevant role of T Helper 17 (Th17) cells in chronic inflammatory and immune‐mediated diseases such as sarcoidosis, contributing to sustained inflammation and granulomatous activity [9].

Furthermore, vascular endothelial growth factor (VEGF) has been implicated in chronic inflammatory diseases, contributing to angiogenesis and tissue remodeling. Although its specific role in sarcoidosis‐like reactions is not fully established, it may play a role in the pathophysiology of granulomatous inflammation [10].

Retrospective studies estimate the incidence of SLRs in patients treated with immunotherapy to range between 0.2% and 7%, depending on tumor type [4, 11]. In melanoma, a potential association between these reactions and improved immune response to treatment has been suggested, although this remains controversial [12]. Some authors propose SLRs as an indirect biomarker of treatment efficacy; however, treatment continuation decisions must be individualized based on toxicity severity.

Management depends on clinical severity. In this case, permanent discontinuation of pembrolizumab was required due to significant multisystem involvement. In mild cases, close monitoring without immunotherapy interruption may be considered, whereas moderate to severe cases generally require temporary or permanent discontinuation and systemic corticosteroid therapy [13]. Rechallenge with immunotherapy should be individualized, with variable outcomes reported [11]. Multidisciplinary management is essential to ensure prompt diagnosis and appropriate management.

The scientific relevance of this case lies in the low incidence of pembrolizumab‐associated SLRs reported in the literature. Most available evidence derives from small case series or isolated reports, and no specific clinical guidelines currently exist for managing these adverse events in routine practice.

It is essential to distinguish between systemic sarcoidosis and SLRs associated with immunotherapy, as the latter are typically drug‐induced, may have a different clinical course, and often require distinct management strategies.

Additionally, the presence of the NRAS Q61L mutation is noteworthy, as NRAS‐mutant melanomas are generally associated with more aggressive behavior and limited response to targeted therapies, although patients may still benefit from immunotherapy [14]. This patient subgroup represents a particular therapeutic challenge, in which the efficacy of immunotherapy may be compromised by the development of irAEs such as the one described. SLRs in melanoma patients are well‐documented, but the published literature has not specifically reported cases in NRAS‐mutant melanoma as a distinct entity. To our knowledge, there is scarce evidence specifically addressing the occurrence of sarcoidosis‐like reactions in this molecular subgroup. Therefore, the potential relationship between NRAS mutation status and irAEs remains unclear and warrants further investigation.

## 4. Conclusions

Sarcoidosis‐like granulomatous reactions represent an uncommon but clinically significant irAE associated with immune checkpoint inhibitors. Early recognition, appropriate differential diagnosis, and multidisciplinary management are essential to avoid unnecessary treatments or premature discontinuation of immunotherapy. Despite their low incidence, these events highlight the need for the development of specific clinical guidelines and further research to better understand their pathophysiology, prognostic impact, and optimal management strategies. Integrating this knowledge into clinical practice is crucial to maximizing the benefits of immunotherapy while ensuring patient safety.

## Funding

No funding was received for this manuscript.

## Ethics Statement

Ethical approval was not required for this case report in accordance with local regulations.

## Consent

Written informed consent was obtained from the patient for publication of this case report and any accompanying images.

## Conflicts of Interest

The authors declare no conflicts of interest.

## Data Availability

All data relevant to the case are included in the article. Additional data are available from the corresponding author upon reasonable request.
